# Longitudinal brain morphology in anti-NMDA receptor encephalitis: a case report with controls

**DOI:** 10.1186/s12888-019-2141-4

**Published:** 2019-05-10

**Authors:** Heikki Laurikainen, Iina Isotupa, Mikko Nyman, Tuula Ilonen, Teija Nummelin, Raimo K. R. Salokangas, Jarmo Hietala

**Affiliations:** 1 Department of Psychiatry, University of Turku and Turku University Hospital, Kunnallissairaalantie 20, Building 9, 20700 Turku, Finland; 2 Turku PET Centre, Kiinanmyllynkatu 4-8, 20521 Turku, Finland; 3 Department of Emergency Radiology, Turku University Hospital, Kiinanmyllynkatu 4-8, 20521 Turku, Finland

**Keywords:** Anti-N-methyl-D-aspartate receptor encephalitis, Non-paraneoplastic, Brain morphometry

## Abstract

**Background:**

Anti-N-methyl-D-aspartate-receptor (NMDAR) encephalitis is a severe autoimmune condition, which typically affects young females. The long-term clinical consequences and brain morphology changes after anti-NMDAR encephalitis are not well known.

**Case presentation:**

We present clinical and neuroimaging follow-up data on a 25-year female patient with typically presenting anti-NMDAR encephalitis. Longitudinal analyses of brain morphology were done using 3 T structural magnetic resonance imaging (sMRI) and Freesurfer analysis at the time of diagnosis and after symptomatic remission. The presented case attained good functional recovery after standard immunoglobulin-corticosteroid treatment but elevated serum NMDAR antibody levels persisted. The patient had no symptomatic relapses during a 3-year clinical follow-up. In the baseline brain sMRI scan there were no marked volume changes. However, a follow-up sMRI after 9 months indicated clear volume reductions in frontal cortical regions compared to matched controls with identical sMRI scans.

**Conclusions:**

This case report of anti-NMDAR encephalitis suggests that despite clinical recovery long-term brain morphological changes can develop in the frontal cortex. Longer clinical and imaging follow-up studies are needed to see whether these frontocortical alterations are fully reversible and if not, can they result in trait vulnerabilities for e.g. neuropsychiatric disorders.

**Electronic supplementary material:**

The online version of this article (10.1186/s12888-019-2141-4) contains supplementary material, which is available to authorized users.

## Background

Anti-NMDAR encephalitis is a type of autoimmune encephalitis associated with the production of autoantibodies against NR1-subunits of NMDA glutamate receptors [1]. It affects predominantly young women and around 40–60% of the cases are associated with a malignancy, most commonly an ovarian teratoma [2]. Despite systematic screening of neoplasms, no clinically detectable tumor can be found in all cases.

The clinical symptomatology of anti-NMDAR encephalitis may vary between patients both in nature and course of symptoms and outcome. The syndrome usually begins with prodromal non-specific flu-like symptoms followed by prominent psychiatric symptoms leading a substantial proportion of patients to be initially evaluated in a psychiatric setting [3]. The symptoms progress into a complex range of psychiatric, neurologic and neuropsychiatric symptoms, such as seizures and catatonia. At worst, prolonged clinical course can be life threatening and require treatment in intensive care unit.

The diagnosis of anti-NMDAR encephalitis requires the detection of NMDAR antibodies in the serum and/or in the spinal fluid (CSF). Cumulative evidence suggests that in most cases clinical brain magnetic resonance imaging (MRI) is normal [4]. Cohort studies have reported MRI abnormalities in 46.5% of adult patients, while abnormal electroencephalography (EEG) is reported in 50–90% with varying findings depending on the stage of the disorder [4–6]. When present, the majority of structural changes are focal lesions of the temporal lobe [4]. General atrophy is less frequent, possibly reversible in nature, and might not reflect symptom severity [4, 7]. While frontotemporal white matter has been shown to be afflicted in patients with poor outcome, frontal lobe atrophy is less frequent, and localized atrophy has been quantified only in the temporal lobe using a cross-sectional design [4, 8–10]. However, robust changes in functional connectivity of large scale brain networks have been observed even when there was no evident pathology in structural MRI [5, 11]. Although associations of structural changes to symptoms severity have been previously described, failure to control for confounding factors, lack of baseline measurements and differences in patient age groups, study design and outcome variable definitions hampers efforts to evaluate overall associations of structural brain changes to functional outcome and/or cognitive symptoms [4, 8, 9, 12–14].

The most important prognostic factors of anti-NMDAR encephalitis are early diagnosis, rapid initiation of immunotherapy and prompt tumor removal. Notably, persistently elevated serum antibodies have been linked to adverse functional prognosis [5, 6]. Recovery is usually slow and patients may relapse, especially if no associated tumor has been found [2, 5, 15]. Cognitive deficits persisting for several years are not uncommon despite effective treatment [16]. Currently proposed first-line treatment options include combinations of tumor removal, intravenous immunoglobulins, plasma exchange and corticosteroids. Recent studies suggest that 53% of patients respond to first-line treatment and up to 47% of patients attain full functional recovery, while 28% recover with mild deficits [2, 6]. Second-line treatment, including pharmacotherapy with rituximab and/or cyclophosphamide, is reserved for patients not responding to first-line treatment. These improve functional outcome and prevent future relapses in patients who did not respond to first-line treatment, [6] or if antibody levels remain elevated despite treatment [17].

The diagnosis and treatment of anti-NMDAR encephalitis still seems to be evolving towards more effective strategies, [11] possibly utilizing modern imaging methods for prompt diagnosis. This trajectory warrants a thorough understanding of disease and treatment effects on brain structure and function, and appreciation of their relevance for outcome. We present here a case of anti-NMDAR encephalitis with good functional outcome despite persistently elevated serum antibody levels. We also demonstrate development of reductions in frontal cortical volumes, while no apparent structural pathology was detectable in conventional neuroradiological assessment.

## Case presentation

A 25-year old woman was admitted to a psychiatric hospital due to psychotic mania. During the preceding month she had experienced elevated mood, decreased sleep, increased thought pressure, excessive spending, distractibility and expansive ideas. One week prior to admission she had been exposed to a traumatic event by proxy, which triggered intense anxiety, loss of energy and motor agitation. She subsequently started experiencing referential thoughts, which were felt as increasingly convincing, and she developed grandiose delusions raising concern in her family. She was escorted for medical and psychiatric evaluation, and was admitted into a psychiatric hospital for treatment. By the time of admission she had also experienced frequent referential delusions to surrounding events, a Fregoli-delusion, visual illusions and auditory speech hallucinations. Her behaviour and speech were disorganized, and affects changed from elation to agitated anxiety.

According to a structured clinical interview for DSM-IV Axis I disorders, the patient was initially diagnosed as suffering from a manic episode with mood congruent psychotic symptoms. Peroral treatment with aripiprazole 15 mg/day and lorazepam 3 mg/day was started. Rorschach comprehensive system revealed sensory distractibility and distorted reality testing [18]. Neuropsychological testing indicated above average performance in psychomotor speed, word fluency, social cognition and working memory and average performance in executive functioning and visual learning performance [19]. Routine clinical and laboratory assessments at admission were normal. There was no evident pathology in brain MRI taken at 29 days from admission. Due to intermittent bouts of low blood pressure, and tachy- or bradycardia, aripirazole was changed to quetiapine 300 mg/day, which was rapidly switched to olanzapine 20 mg/day. Olanzapine was then combined with lithium 300 mg/day due to increasingly volatile affects, novel paranoid delusions and an apparent lack of therapeutic effect.

Elbow rigidity, gait abnormalities and tremors had been absent at admission. After 1 month of antipsychotic treatments olanzapine was discontinued due to difficulties in focusing vision, joint rigidity and shuffling gait. Extrapyramidal and motor symptoms continued to worsen nevertheless. Joint rigidity increased gradually, and at the same time the patients speech became slower, verbal content poor, and affects blunted. The poor clinical response to antipsychotics prompted a neurology consultation. An EEG examination was normal but oligoclonal bands of IgG were found in cerebrospinal fluid (CSF) when blood and CSF samples for autoimmune encephalitis were drawn 8 weeks after admission to psychiatric hospital. Mood stabilizing medications were discontinued as neurologic symptoms worsened with opisthotonus posturing, retrocollis, swallowing difficulties, increased salivation, nocturnal incontinence, autonomic instability and periodic unresponsiveness. Malignant neuroleptic syndrome was previously ruled out in an emergency consultation. In the context of fluctuating psychotic, manic and disorganized behavior, a clinical picture of catatonia started to emerge including mutism, mannerisms and incoherence. The patient received a series of three sessions of electroconvulsive therapy without clinical response.

At this time, 82 days after admission to psychiatric hospital, the serum and CSF sample results for NMDA receptor antibodies were reported as positive. IgG antibodies were detected using indirect immunofluorescence of 1:10 diluted serum or undiluted CSF incubated on cerebellar tissue (Clinical Immunological Laboratory, Prof. Dr. med. Winfried Stöcker, Lübeck, Germany). The patient was transferred to a neurology ward and intravenous (i.v.) methylprednisolone 1 g/day and i.v. immunoglobulins 20 g/day were started and commenced for 5 days. Peroral lorazepam was continued at 4 mg/day, and prednisone was started perorally after i.v. treatments at 50 mg/day. Ovarian teratomas and tumors were excluded with a pelvic MRI and whole body computer tomography. Positive titers of serum NMDA receptor antibodies were still measured at 166 days and 242 days after admission to psychiatric hospital. One infusion of rituximab 500 mg i.v. was given at 220 days due to persistently elevated antibody titers.

The premorbid level of functioning of our patient had been excellent, with a highest global assessment of functioning (GAF) level of 91 during the past year. At the time of admission to psychiatric hospital GAF was 43, and at the start of neurologic rehabilitation GAF was 21. Prior to illness, the patient had been working to complete a university degree after a lifetime history of academic success at international schooling. Prejuvenile and juvenile social adjustments were good, [20] and there were no history of neurological illness, chronic medical illness, perinatal complications, head trauma, nor history of psychiatric disorders or substance use disorders in the family.

After 50 days of treatment in the neurological ward the patient was discharged. She received 15 sessions of neuropsychological rehabilitation, speech therapy and psychological counseling during outpatient treatment and further functional recovery was gained. Disregarding subjective concentration difficulties, objectives for neuropsychological recovery were attained. Medical status was assessed again at 290 days after admission to psychiatric hospital. All medications had been discontinued 3 months prior to this assessment. The patients weight and abdominal circumference changed marginally between assessments at 14 and 290 days from 55 kg to 54 kg, and 78 cm to 76 cm respectively. At this time GAF was rated at 70, which was the highest level of functioning during the past year. She had only mild subjective experiences of difficulties in verbal expression, distractibility, concentration difficulties, slowed-down thinking and lack of thought energy. Auditory working memory performance was solely reduced in repeated neuropsychological testing compared to baseline, but remained within normal limits. Positive symptoms, catatonia, joint rigidity or gait disturbances were no longer present. There was no detectable pathology or longitudinal change in conventional brain MRI. Serum NMDAR antibody titers were still positive at 408 days from admission The patient has subsequently completed her university studies, and has been since employed in full time work. All subjective neuropsychological symptoms have disappeared and no symptomatic relapses have occurred during a 946 day follow-up.

We further wanted to explore for subclinical morphological changes between the first and latest brain T1 weighted MRI using Freesurfer image analysis suite (http://surfer.nmr.mgh.harvard.edu/) for cortical reconstruction and volumetric segmentation in a longitudinal design [21]. The patient, and eight healthy female controls of similar age, underwent the same structural MRI scan with the Philips 3 T Ingenuity PET/MR hybrid scanner at baseline, and after 9 months with same imaging sequences. We compared regional volume changes between time points with changes measured from the reference sample. Values deviating from the lower or upper quartile for more than 1.5 times the interquartile range (IQR) was chosen as a cutoff for outlier status from reference population. See Additional file 1: Supplementary methods for details.

**Fig. 1:**
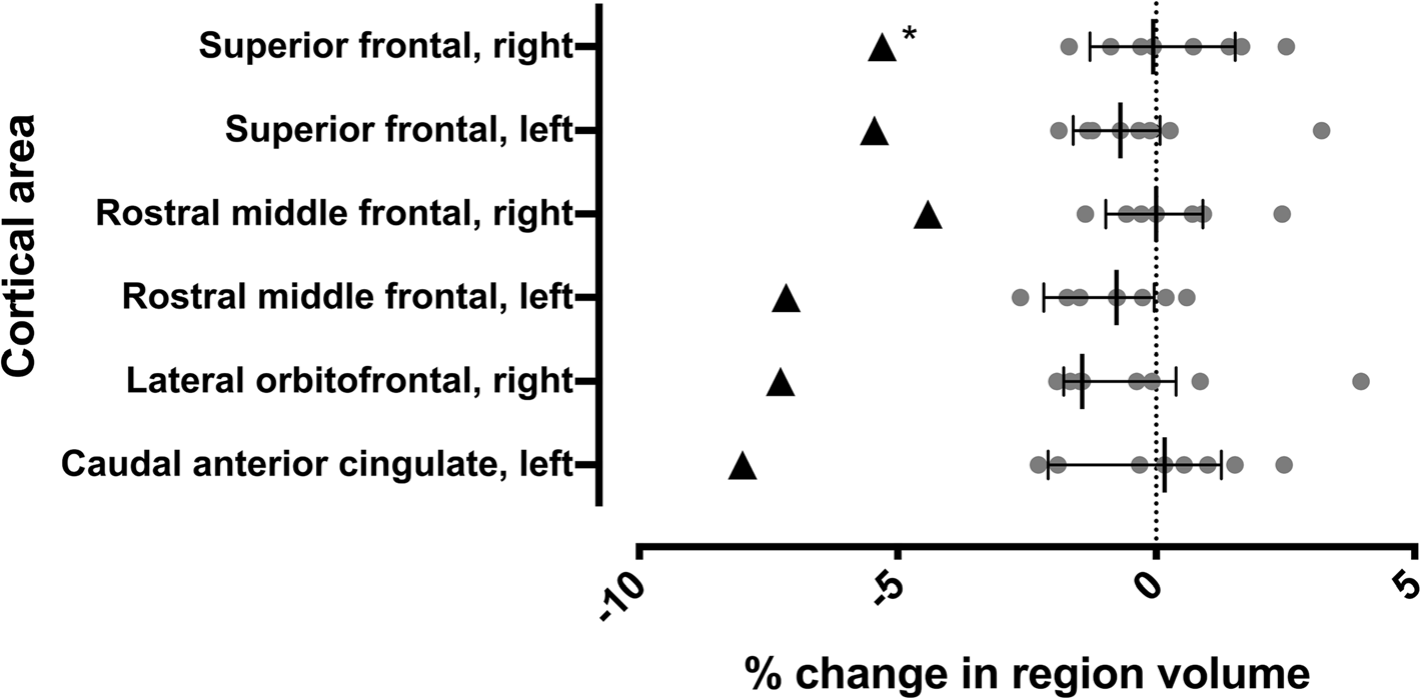
Proportional change of cortical region volume from baseline to 9-month follow-up period. The case is denoted by black triangles (▲), while grey dots (●) are reference subjects. The median value of all subjects is denoted by a vertical black line. Error bars represent the upper and lower quartiles. *: cortical volume reduction = 1.42xIQR

In the baseline MRI scan of this case conventional neuroradiological assessment revealed no major pathology. A more detailed volumetric analysis revealed lower volumes in multiple brain regions, but these did not clearly deviate from the control subjects when corrected for intracranial volume. However, the patient had more volume reduction between baseline and the follow-up time point than any subject in the reference group in primarily frontal cortical regions (Fig. 1). Movement artefact was minimal, but slightly more prominent in the patient baseline MRI scan compared to the follow-up scan. Movement during a functional MRI series from the same scan session was higher in baseline for the patient, but there was no association of change in movement to change in volume in the complete study sample.

## Discussion and conclusions

In a previous study the functional outcome of anti-NMDAR encephalitis was invariably associated to decline in serum antibody levels after treatment [2]. Notably, in this case constantly positive serum antibody titers, or changes in regional brain volumes, were not related to poor functional outcome or residual neuropsychological consequences. We used the very same MRI imaging protocol and 3 T MRI scanner and took into account the movement bias of MRI volumetric analysis. We could not find an obvious association of movement to volume in this sample. At most, the direction of association compared to previous findings in these cortical areas would be reversed, suggesting that the volume changes are unlikely to be explained by head movement in scanner [22]. Because the reference group was of similar sex and age and there were no outliers regarding movement, the cause of extreme deviations in volume change are interpreted here to result from differing source populations: the population of anti-NMDAR encephalitis patients compared to healthy individuals.

Longitudinal volume reductions despite no findings in routine radiological assessment suggests that subtle brain pathology can be measured, and putatively even used for clinical decision making. Such neuropsychiatric consequences of brain inflammation, could manifest later as problems in brain maturation and vulnerabilities to psychiatric symptoms, e.g. through functional dysconnectivity or altered cortical excitation/inhibition balance. These changes might be even more pronounced in the developing brain at a younger age. Although the results from one case cannot be generalized further, our findings raise important questions about the longitudinal consequences of autoimmune encephalitis, the role of conventional radiological imaging in monitoring progression, and the relevance of peripheral antibody levels for functional outcome.

The volumetric changes could also be due to psychiatric or immunological medication effects. All antipsychotic, mood stabilizing and anxiolytic medications in this case were discontinued respectively 8, 7 and 3 months prior to follow-up MRI evaluation, and the immediate therapeutic effect of the first two treatment strategies were evaluated as insufficient. Thus, any direct contribution of the psychiatric medications to MRI finding is unlikely. Also, it seems improbable that electroconvulsive therapy would have contributed since the received dose was well below suggested therapeutic levels, electroconvulsive therapy has been shown to be associated with volume increases rather than volume loss, [23, 24] and lastly the follow-up MRI was taken over 6 months after the last dose. In this case rituximab, a medication with potential for severe adverse effects, was administered on the basis of persistently elevated antibody levels. The complete functional recovery despite elevated antibody levels suggests a very modest effect of rituximab treatment at least in this patient. Any adverse iatrogenic effects would implicate a need for more accurate measures of outcome, and given that many anti-NMDAR encephalitis patients are initially treated in a psychiatric setting, more sensitive differential diagnostic tools for psychotic syndromes.

This case of typically presenting anti-NDMA receptor encephalitis attained good functional recovery despite persistently elevated serum antibody levels. We were able to detect long-term volume reductions in frontal cortical areas at 9 month follow-up, which suggests that the utility of more sensitive volumetric measurements might be worthwhile to investigate also in the clinical context. Studies with larger sample sizes are needed to assess the generalizability of our observations, while longitudinal study designs with very long follow-ups would clarify the trajectory of brain volume changes and their relationships between relapse risk, neuropsychiatric sequelae and functional outcome in anti-NMDAR encephalitis.

## Additional file

Additional file 1: Supplementary methods. (DOCX 113 kb)

## Abbreviations

CSF: Cerebrospinal fluid
DSM: Diagnostic and statistical manual for mental disorders
EEG: Electroencephalography
GAF: Global assessment of functioning
i.v: intravenous
IgG: Immunoglobulin type G
IQR: Inter-quartile range
MRI: Magnetic resonance imaging
NMDAR: Anti-N-methyl-D-aspartate-receptor
sMRI: structural magnetic resonance imaging
