# Supplementary material for: Longitudinal brain morphology in anti-NMDA receptor encephalitis: a case report with controls
Source: BMC Psychiatry. 2019 May 10;19:145. doi: 10.1186/s12888-019-2141-4 (PMC6511133; doi:10.1186/s12888-019-2141-4)
Supplement: Supplementary file 1 — Supplementary methods. (DOCX 113 kb) [file 12888_2019_2141_MOESM1_ESM.docx]

Additional file 1

# SUPPLEMENTARY METHODS

We included eight healthy controls matched for age and sex to act as a reference group for the volumetric analyses. All subjects were scanned with a 3 Tesla Philips Ingenuity PET/MR scanner using a T1-weighted sequence (Ultrafast Gradient Echo 3D, TR=8.1 ms, TE-time=3.7 ms, flip angle=7°, FOV=256x256x176 mm^3^ and voxel size=1x1x1 mm^3^). All T1 weighted scans were repeated until movement artefact was minimal. No scans were excluded due to poor image quality as assessed radiologically. Structural abnormalities were ruled out by a neuroradiologist. To assess for bias in volumetric analyses movement artefact was evaluated visually and graded as being decreased, similar or higher in baseline compared to follow-up. The direction of association between scan movement and volumetric indices in Freesurfer longitudinal analyses has been previously described (Reuter et al., 2015). Additionally, transformations parameters obtained from spatial re-alignment of an fMRI series, from the same scanning session, were used to calculate a mean and maximum movement indice during scan. Spatial re-alignment was done with SPM12 and Matlab 2014b (The Mathworks Inc., Sherborn, Massachusetts).

Cortical reconstruction and volumetric segmentation was performed with the Freesurfer image analysis suite, which is documented and freely available for download online (<http://surfer.nmr.mgh.harvard.edu/>). The technical details of these procedures are described in prior publications. To extract reliable volume and thickness estimates of differences between timepoints, images were automatically processed with the longitudinal stream (Reuter et al., 2012) in FreeSurfer. Descriptive statistics were done with IBM SPSS Statistics 23 (IBM corp. Armonk, NY, USA) and Graphpad Prism 4 (GraphPad Software, San Diego California USA). Due to the small size of the reference sample, robust measures of median and interquartile range (IQR) were chosen as the location indicator and measure of statistical dispersion of proportional volume changes. Values deviating from the lower or upper quartile for more than 1.5x the interquartile range (IQR) was chosen as a cutoff for outlier status from reference population.
